# Effect of different surgical procedures on the accuracy of prediction of the plasma concentration of fentanyl: comparison between mastectomy and laparoscopic prostatectomy

**DOI:** 10.1186/s40981-017-0097-2

**Published:** 2017-05-19

**Authors:** Yoshihito Fujita, Saya Yoshizawa, Maiko Hoshika, Koichi Inoue, Shoko Matsushita, Hisao Oka, Kazuya Sobue

**Affiliations:** 1 Department of Anesthesiology, Aichi Medical University School of Medicine, 1-1 Karimata Yazako, Nagakute, Aichi 480-1195 Japan; 2 Department of Anesthesiology and Intensive Care Medicine, Nagoya City University Graduate School of Medical Sciences, 1 Kawasumi, Mizuho-cho, Mizuho-ku, Nagoya, Japan; 3 Department of Physical and Analytical Chemistry, School of Pharmacy, Kinjo Gakuin University, 2-1723 Omori, Moriyama, Nagoya, 463-8521 Japan

**Keywords:** Fentanyl, Concentration, Simulation, Prediction

## Abstract

**Background:**

The accuracy of simulation-predicted fentanyl concentration in different types of surgical procedure is not fully understood. We wished to estimate the effect of different types of surgical procedure on the accuracy of such simulations.

**Findings:**

Fifty patients who had undergone elective mastectomy or laparoscopic prostatectomy (American Society of Anesthesiologists physical status = I–II) were enrolled. Anesthesia was maintained throughout surgery with sevoflurane and a bolus infusion of fentanyl. A maintenance infusion was administered with 8 mL/kg/h Ringer’s acetate solution from the start of anesthesia to completion of blood sampling. An infusion to compensate for blood loss was administered (one to two volumes of hydroxyethyl starch). A blood sample was drawn every 30 min during anesthesia.

We measured the plasma concentration of fentanyl in 358 samples from 50 patients. The plasma concentration of fentanyl was correlated significantly with the simulated predicted fentanyl concentration (*r* = 0.734, *P* < 0.01) but 36.0% of all samples had a difference greater than ±0.5 ng/mL. Approximately 0.3 ng/mL of a fixed bias was shown throughout mastectomy. During laparoscopic prostatectomy, the fixed bias gradually became negative from ≈0.3 to −0.3 ng/mL as the sampling stage proceeded.

**Conclusions:**

The predicted concentration of fentanyl was significantly correlated with the plasma concentration of fentanyl (*r* = 0.734). However, there were different patterns of a fixed bias between mastectomy and laparoscopic prostatectomy groups. We should pay attention to this tendency among different surgical procedures.

**Trial registration:**

UMIN000005110

**Electronic supplementary material:**

The online version of this article (doi:10.1186/s40981-017-0097-2) contains supplementary material, which is available to authorized users.

## Findings

### Introduction

Fentanyl is a synthetic opioid used widely to supplement general anesthesia. Fentanyl is administered in general anesthesia upon prediction of its concentration with a simulator. The Shafer model is used worldwide [1]. This predictive model is applied for various types of surgical procedure. Recently, interest has focused on which other factors may be responsible for alterations in pharmacokinetics with the objective of reducing variability and increasing accuracy. Kazama and colleagues, using a pseudo-steady-state model of propofol, found that compensated hemorrhage increased the propofol concentration by 20%; however, if uncompensated shock was induced, the propofol concentration increased by 375% [2]. Using a non-steady-state model, Egan and colleagues found that hemorrhagic shock reduced central clearance as well as the central volumes of fentanyl [3] and remifentanil, resulting in a higher fentanyl concentration in animals suffering from shock. Other factors, such as age [4, 5], sex [6, 7], cardiac output [8, 9], and obesity [10], were also investigated.

However, the accuracy of the simulator in different surgical procedures is not fully understood. In the present study, we chose mastectomy and laparoscopic prostatectomy because the former has a short operative time and low blood loss, whereas the latter has a relatively long operative time and several hundred blood loss. We investigated the effect of these different surgical procedures on the accuracy of simulation-predicted fentanyl concentration in plasma.

### Materials and methods

Approval (number 528) of the study protocol by the Research Ethics Board was obtained, as was written informed consent from patients. This study is registered with the University Hospital Medical Information Network Clinical Trials Registry (UMIN-CTR, UMIN000005110).

Patients graded as class I or II according to the classification set by the American Society of Anesthesiologists undergoing mastectomy or laparoscopic prostatectomy were enrolled. Patients with the following conditions were excluded: (i) American Society of Anesthesiologists physical status class III, IV, or V; (ii) age <19 or >71 years; (iii) body mass index >30 kg/m^2^; (iv) height >181 cm; and (v) weight >80 kg.

Anesthesia was induced with propofol (1.5–2.0 mg/kg body weight), fentanyl (1–3 μg/kg), and a bolus infusion of rocuronium (1 mg/kg). Anesthesia throughout the surgical procedure was maintained with sevoflurane and fentanyl after a bolus infusion of propofol and rocuronium. After the initial dose of fentanyl, a bolus infusion of fentanyl (25, 50, or 100 μg/bolus) was adjusted by the participating anesthesiologist to meet the clinical need in each situation. Anesthesia was titrated subsequently to a bispectral index of ≈50. Continuous intravenous infusion of Ringer’s acetate solution was started in the operating room. A maintenance infusion was administered with 8 mL/kg/h Ringer’s acetate solution from the start of anesthesia until blood samples had been obtained. In addition, infusion for blood loss was administered with one to two volumes of 6% hydroxyethyl starch (70/0.5). Pharmacokinetic simulations were not used to guide anesthesia: they were carried out after the cases had been completed.

Blood samples from an arterial catheter were taken every 30 min from the initial administration of fentanyl to 30 min after the end of the surgical procedure. Samples were placed immediately on ice and centrifuged at 1000 × *g* and 4° for 5 min within 1 h, and the plasma was stored at −80 °C until analyses. Samples taken within 10 min of the bolus infusion of fentanyl were excluded from analyses.

Quantification of fentanyl in plasma was done using online solid-phase extraction liquid chromatography coupled with mass spectrometry (Micromass Quattro Premier; Waters, Milford, MA, USA) in multiple-reaction monitoring mode [11]. The monitoring ions were *m/z* 337.1 → 187.9 for fentanyl and *m/z* 342.1 → 187.9 for fentanyl-d_5_ (internal standard), respectively. The lower limit of quantitation of fentanyl in human plasma was 0.05 ng/mL. Recovery values ranged from 99 to 111% for inter-day (relative standard deviation: 1.7–6.9%) and intra-day assays (1.0–10.1%). This method was applied for measurement of the fentanyl level in plasma from patients.

The pharmacokinetic model described by Shafer was used to predict the plasma concentration of fentanyl. In conjunction with the administration schedule for each patient, the Tivatrainer program (www.eurosiva.eu/tivatrainer/TTweb/TTinfo.html) was used to generate the predicted fentanyl concentration in plasma for the Shafer model [1].

The present study was designed to detect a correlation between the predicted and measured concentrations of fentanyl in plasma. The effect size was a correlation coefficient (*r*) >0.5. Power analyses indicated that a minimum of 38 samples would be needed to detect this correlation with a power of 90% and an α of 0.05. We estimated that, to analyze three to four subgroups, we would need ≈160 samples (38 multiplied by 4) for the mastectomy and laparoscopic prostatectomy groups. Continuous variables are presented as the mean ± SD or median (interquartile range) depending on the normality of the distribution as confirmed by the Shapiro–Wilk test.

### Results

Fifty patients were recruited between February and November 2011. Thirty patients undergoing mastectomy and 20 patients undergoing laparoscopic prostatectomy completed the study with 358 usable data. The cardiac, renal, and liver functions of all patients were normal. The fentanyl concentration in the plasma of 410 samples was measured. However, 48 samples of control data from before fentanyl addition and four samples taken within 10 min of the bolus infusion of fentanyl were excluded because of inappropriate timing. Baseline characteristics are shown in Table 1.

**Table 1:** Characteristics of the patients

	Mastectomy	Laparoscopic prostatectomy
	Number = 30	Number = 20
Age (years)	52.3 ± 9.7	66 (63, 70) [45, 70]
Sex	Female	Male
Height (cm)	156.6 ± 5.0	168.3 ± 6.5
Weight (kg)	52.9 ± 7.7	64.1 ± 9.3
AHA physical status I, II	22, 8	8, 12
Operation time (min)	115.1 ± 35.9	179.5 (163, 300) [139, 344]
Anesthesia time (min)	160.8 ± 39.5	251.5 (219, 364.5) [195, 407]
Fentanyl (μg)	566.0 ± 169.0	900 (812.5, 900) [500, 1700]
Number of samples	176	182
Infusion		
AR	941.3 ± 381.1	1750 (1525, 2465) [1050, 3200]
HES	0 (0, 0) [0, 1000]	600 (425, 1650) [200, 2700]
Blood loss	24 (5,44) [5, 679]	563.5 (400, 739) [52, 2550]^a^

^a^Blood loss in laparoscopic prostatectomy includes urine from the surgical field

*AR* acetate Ringer’s solution, *HES* hydroxyethyl starch

**Fig. 1:**
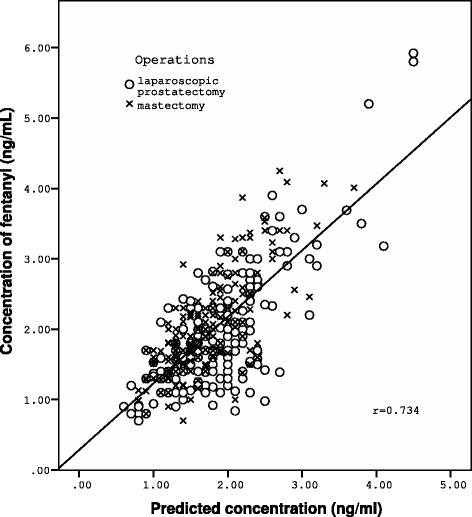
Correlation between the plasma concentration of fentanyl and simulation-predicted concentration of fentanyl. The plasma concentration of fentanyl was correlated significantly with the simulation-predicted concentration of fentanyl (*r* = 0.734, *P* < 0.01, *y* = 0.94x + 0.29)

**Fig. 2:**
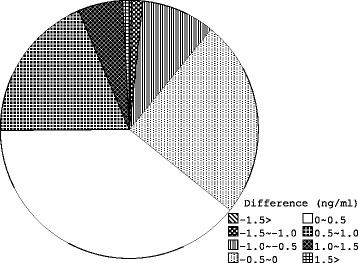
Difference in the fentanyl concentration between plasma and simulation for 358 samples. Among these samples, 36.0% had a difference of greater than ±0.5 ng/mL

**Fig. 3:**
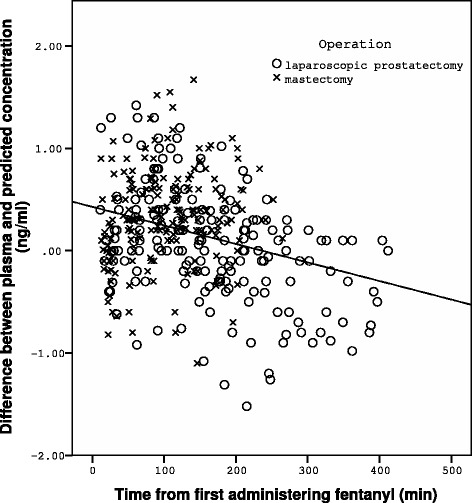
Difference between the plasma concentration of fentanyl and simulation-predicted concentration of fentanyl plotted against time from the first administration of fentanyl. There was an effect of time from the first administration of fentanyl on the difference between the plasma concentration of fentanyl and simulation-predicted fentanyl concentration (*r* = −0.293, *P* < 0.01, *y* = −0.02x + 0.429)

The correlation between the plasma concentration of fentanyl and predicted plasma concentration of fentanyl is shown in Fig. 1. The plasma concentration of fentanyl was correlated significantly with the predicted concentration (*r* = 0.734, *P* < 0.01, *y* = 0.94x + 0.29). The difference in the fentanyl concentration between plasma and the simulation for the 358 samples is shown in Fig. 2. The difference between the plasma concentration of fentanyl and simulation-predicted fentanyl concentration was 0.19 ± 0.54 [range, −1.52 to 1.67]. In addition, 36.0% of all samples had a difference of more than ±0.5 ng/mL. The difference between the plasma concentration of fentanyl and simulation-predicted fentanyl concentration plotted against time from the first administration of fentanyl is shown in Fig. 3. There was an effect of time from the first administration of fentanyl on the difference between the plasma concentration of fentanyl and simulation-predicted fentanyl concentration (*r* = −0.293, *P* < 0.01, *y* = −0.02x + 0.429).

**Fig. 4:**
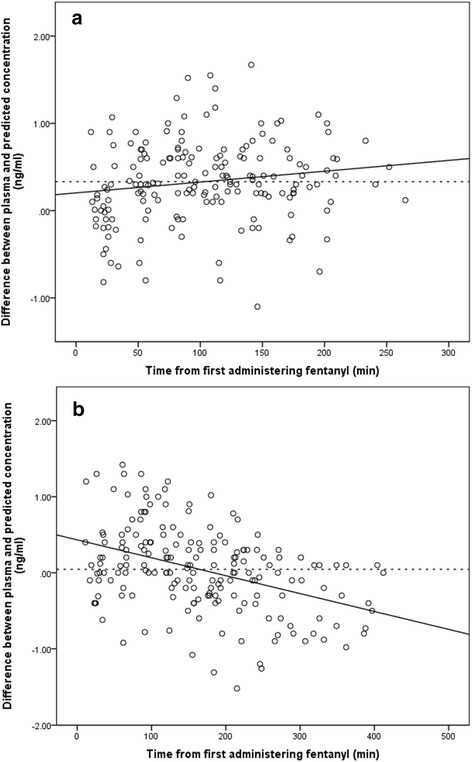
Difference in the predictive accuracy between mastectomy and laparoscopic prostatectomy. In mastectomy, the difference between the measured concentration of fentanyl and simulation-predicted concentration of fentanyl had no correlation with time from the first administration of fentanyl (*r* = 0.157, *P* < 0.01, *y* = 0.01x + 0.203). Approximately 0.3 ng/mL of a fixed bias was shown in all sampling stages (**a**). However, in the laparoscopic prostatectomy group, a fixed bias was influenced by sampling stage (**b**), and gradually became negative as the sampling stage increased. This finding implies that ≈0.3 ng/mL of a fixed bias in stage 1 decreased to −0.3 ng/mL in stage 4 systematically (*r* = −0.411, *P* < 0.01, *y* = −0.02x + 0.432)

We wished to examine the relationship between the different surgical procedures and the influence of different sampling times. Hence, sampling data were divided into four groups at 90-min intervals for each type of surgical procedure. Samples of arterial blood were obtained within 1.5 h from the initial administration of fentanyl (stage 1), during 1.5 to 3 h (stage 2), during 3 to 4.5 h (stage 3), and after 4.5 h (stage 4). Interestingly, our data showed that the tendency of a fixed bias differed between mastectomy and laparoscopic prostatectomy. In mastectomy, the difference between the measured fentanyl concentration and simulation-predicted fentanyl concentration had no correlation with time from the first administration of fentanyl (*r* = 0.157, *P* < 0.01, *y* = 0.01x + 0.203). Approximately 0.3 ng/mL of a fixed bias was shown in all sampling stages (Fig. 4a). However, in laparoscopic prostatectomy, a fixed bias was influenced by the sampling stage (Fig. 4b) and gradually became negative as the sampling stage increased. More details are shown in Additional file 1.

### Discussion

In the present study, we demonstrated that the predicted data were significantly correlated with the plasma concentration of fentanyl (*r* = 0.734). However, a different pattern of a fixed bias appeared between mastectomy and laparoscopic prostatectomy. In mastectomy, ≈0.3 ng/mL of a fixed bias was shown in all sampling stages. However, in laparoscopic prostatectomy, a fixed bias became increasingly negative from ≈0.3 to −0.3 ng/mL as the sampling stage proceeded. Hence, anesthesiologists should pay attention to this difference among different surgical procedures.

In a clinical setting, the primary concern has been how far the measured concentration is from the predicted concentration [1]. The accuracy of simulation has been described most frequently in terms of the “performance error.” That is, the difference between the measured concentration and target concentration as a percentage of the desired target (i.e., [measured − target]/target × 100%) [12]. However, in the present study, the difference in the fixed bias due to the type of surgical procedure revealed that the median absolute performance error did not show this tendency (Table 2). In a clinical setting, the accuracy of the performance error might not be sufficient to detect a difference between the predicted fentanyl concentration and measured fentanyl concentration.

**Table 2:** Analyses of each sampling stage

Sampling stage	Stage 1	Stage 2	Stage 3	Stage 4
Sampling time	<1.5 h	1.5–3 h	3–4.5 h	>4.5 h
Mastectomy				
Number of samples	83	74	19	0
Plasma fentanyl concentration (ng/mL)	2.08 ± 0.74	2.07 ± 0.70	1.88 ± 0.54	
Simulated fentanyl concentration (ng/mL)	1.81 ± 0.54	1.68 ± 0.52	1.50 ± 0.31	
The difference between plasma and predicted fentanyl	0.27 ± 0.48	0.38 ± 0.47	0.34 ± 0.44	
Range of difference	[−0.82, 1.52]	[−1.1, 1.68]	[−0.7, 1.1]	
Number of a difference more than +0.5 ng/mL	28/83 (38%)	27/74 (36%)	6/19 (32%)	
Number of a difference less than −0.5 ng/mL	6/83 (7%)	3/74 (4%)	1/19 (5%)	
Median absolute performance error (%)	21	25	35	
Fixed bias [95% confidence interval] (ng/mL)	0.27 [0.17 0.37]	0.38 [0.27 0.49]	0.38 [0.17 0.59]	
Laparoscopic prostatectomy				
Number of samples	48	59	48	27
Plasma fentanyl concentration (ng/mL)	2.27 ± 1.26	2.08 ± 0.63	1.68 ± 0.56	1.43 ± 0.43
Simulated fentanyl concentration (ng/mL)	1.98 ± 1.04	1.90 ± 0.47	1.83 ± 0.55	1.76 ± 0.48
The difference between plasma and predicted fentanyl	0.29 ± 0.54	0.19 ± 0.52	−0.15 ± 0.53	−0.34 ± 0.41
Range of difference	[−0.92, 1.42]	[−1.08, 1.20]	[−1.52, 0.78]	[−0.98, 0.30]
Number of a difference more than +0.5 ng/mL	13/48 (27%)	14/59 (26%)	2/48 (4%)	0/27 (0%)
Number of a difference less than −0.5 ng/mL	2/48 (4%)	5/59 (8%)	10/48 (21%)	12/48 (25%)
Median absolute performance error (%)	18	18	20	21
Fixed bias [95% confidence interval] (ng/mL)	0.29 [0.14 0.44]	0.18 [0.03 0.33]	−0.15 [0 −0.30]	−0.34 [−0.17 −0.51]

There were differences between mastectomy and laparoscopic prostatectomy in terms of patient age, sex, operative time, blood loss, volume of infusion, and other factors (Table 1). One of the reasons for the difference in the volume of infusion might have been dilution of the administered fluid compensating for blood loss. Blood loss in laparoscopic prostatectomy was greater than that in mastectomy (Table 1). In our protocol, the maintenance infusion remained constant, and infusion for blood loss was administered with one to two volumes of hydroxylethyl starch according to the clinical strategy. We considered that increasing the infusion for blood loss might have caused a reduction in the plasma concentration of fentanyl. Several authors have concluded that hemorrhage increases the drug concentration [2, 3]. Those results are not in accordance with the results in the present study. In our study, patients were administered a sufficient volume to avoid hemorrhagic shock. In addition, we assumed that, in those studies, hemorrhagic shock itself induced a reduction in fentanyl clearance, for example, by reducing central clearance [3]. Without shock status, the fentanyl concentration might be influenced directly by dilution of the infusion.

Several studies have investigated the effect of age on pharmacokinetic parameters [4, 5]. Pharmacokinetic parameters change in the elderly, but whether these changes are sufficiently different to affect the accuracy of measurement of these parameters has not been established. In our study, the patients in the laparoscopic prostatectomy group were older than those in the mastectomy group. In general, elderly patients may be considered to have a lower clearance of fentanyl. In addition, this scenario might cause a higher concentration of fentanyl during laparoscopic prostatectomy. Nevertheless, lower concentrations compared with simulation were shown during laparoscopic prostatectomy. This factor might not have influenced the results of this study. We considered that the age difference might not have affected the outcomes of our study markedly.

Cardiac output has been shown to affect the early pharmacokinetics of fentanyl distribution [8, 9]. Higher cardiac output has been shown to reduce the arterial concentration of fentanyl compared with that in a model of normal cardiac output [9]. Especially in the laparoscopic prostatectomy group, the administration of hydroxyl ethyl starch for the compensation of blood loss might have increased cardiac output and influenced our results.

The intensity of nociceptive stimulation during a surgical procedure may change the pharmacokinetics of a drug because pain induces vascular contraction via sympathetic nerves. The intensity of nociceptive stimulation might have been different between mastectomy and laparoscopic prostatectomy. Nevertheless, mastectomy and laparoscopic prostatectomy are not strongly invasive types of surgical procedure. Therefore, this factor might not have affected the results of this study markedly.

Our investigation had two main limitations. First, we chose patients who had undergone mastectomy or laparoscopic prostatectomy. The data obtained did not cover simulations of all types of surgical procedure. In addition, using data from different types of surgical procedure besides mastectomy and laparoscopic prostatectomy would have been beneficial. Second, opioid doses are often based on the total body weight of a patient. In the present study, the total body weight of almost all patients was in the normal range (Table 1). Thus, our data cannot be extrapolated to lean or obese patients.

### Conclusion

We demonstrated a variation in the difference between the plasma concentration of fentanyl and predicted concentration of fentanyl in a clinical setting. The predicted concentration of fentanyl was significantly correlated with the plasma concentration of fentanyl, with *r* = 0.734. However, there were several patterns of fixed bias between the mastectomy and laparoscopic prostatectomy groups. Our results suggest that the type of surgical procedure should be considered when predicting the concentration of an anesthetic agent.

## Additional file

Additional file 1: Supplementary files. (ZIP 97 kb)
