# Supplementary material for: Effect of different surgical procedures on the accuracy of prediction of the plasma concentration of fentanyl: comparison between mastectomy and laparoscopic prostatectomy
Source: JA Clin Rep. 2017 May 19;3:30. doi: 10.1186/s40981-017-0097-2 (PMC5804613; doi:10.1186/s40981-017-0097-2)
Supplement: Supplementary file 1 — Supplementary files. (ZIP 97 kb) [file 40981_2017_97_MOESM1_ESM.zip › additional file 1.docx]

Additional file 1,

**The different tendency of a fixed bias between mastectomy and laparoscopic prostatectomy.**

To examine the relationship between the different operations and the influence of different sampling times, sampling data were divided into 4 groups for 90-minute intervals in each surgery. Arterial blood samples were obtained within 1.5 h from the initial fentanyl administration (Stage 1), during 1.5 h to 3 h (Stage 2), during 3 h to 4.5 h (Stage 3), and after 4.5 h (Stage 4). Interestingly, our data showed that the tendency of a fixed bias differed between mastectomy and laparoscopic prostatectomy. In mastectomy, the difference between measured and simulation predicted concentration had no correlation with time from the first administration of fentanyl (r=0.157, P<0.01, y= 0.01x+0.203). Approximately 0.3 ng/ml of fixed bias was shown in all sampling stages (Figure 4A). However, in laparoscopic prostatectomy, fixed bias was influenced by sampling stage (Figure 4B). It gradually became negative as the sampling stage increased. This implies that approximately 0.3 ng/ml of fixed bias in stage 1 decreased to -0.3 ng/ml in stage 4, systematically (r= -0.411, P<0.01, y= -0.02x+0.432). Underestimation of the predicted fentanyl concentration thus gradually changed to overestimation as surgery proceeded. In addition, in each group, the mean differences, the number of differences greater than ±0.5 ng/ml, and the median absolute performance error (MDAPE) were investigated and are listed in Table 2.

The above tendency was confirmed by the mean difference between plasma and simulation predicted fentanyl concentrations and the number of differences greater than ±0.5 ng/ml (Table 2). In mastectomy, in all stages, the difference between plasma and simulation predicted fentanyl concentrations were consistently from 0.27 to 0.38 ng/ml. The percentage of differences greater than +0.5 ng/ml was 32-38% and the percentage of differences less than –0.5ng/ml was 4-7%. However, in laparoscopic prostatectomy, as surgery proceeded, the difference between plasma and simulation predicted fentanyl concentrations changed from 0.29 to -0.39. The percentage of differences greater than +0.5 ng/ml decreased from 27% to 0% and the percentage of differences less than –0.5 ng/ml increased from 4% to 25%, gradually. The time courses of all patients’ differences between predicted and measured fentanyl concentrations are shown in Figure 5, in which the above tendency can be seen.

**Figure Legends**

**Figure 5:** Time course of differences between predicted and measured fentanyl concentrations of all patients. Figures A 1-3 are of the 30 patients with mastectomy and Figures B 1 and 2 are of the 20 patients with laparoscopic prostatectomy. Horizontal axis shows the time course (min). Vertical axis shows the difference between predicted and measured fentanyl concentrations (ng/ml).
