# Supplementary material for: Effect of different surgical procedures on the accuracy of prediction of the plasma concentration of fentanyl: comparison between mastectomy and laparoscopic prostatectomy
Source: JA Clin Rep. 2017 May 19;3:30. doi: 10.1186/s40981-017-0097-2 (PMC5804613; doi:10.1186/s40981-017-0097-2)
Supplement: Supplementary file 1 — Supplementary files. (ZIP 97 kb) [file 40981_2017_97_MOESM1_ESM.zip › additional file 2.pdf]

## Slide 1
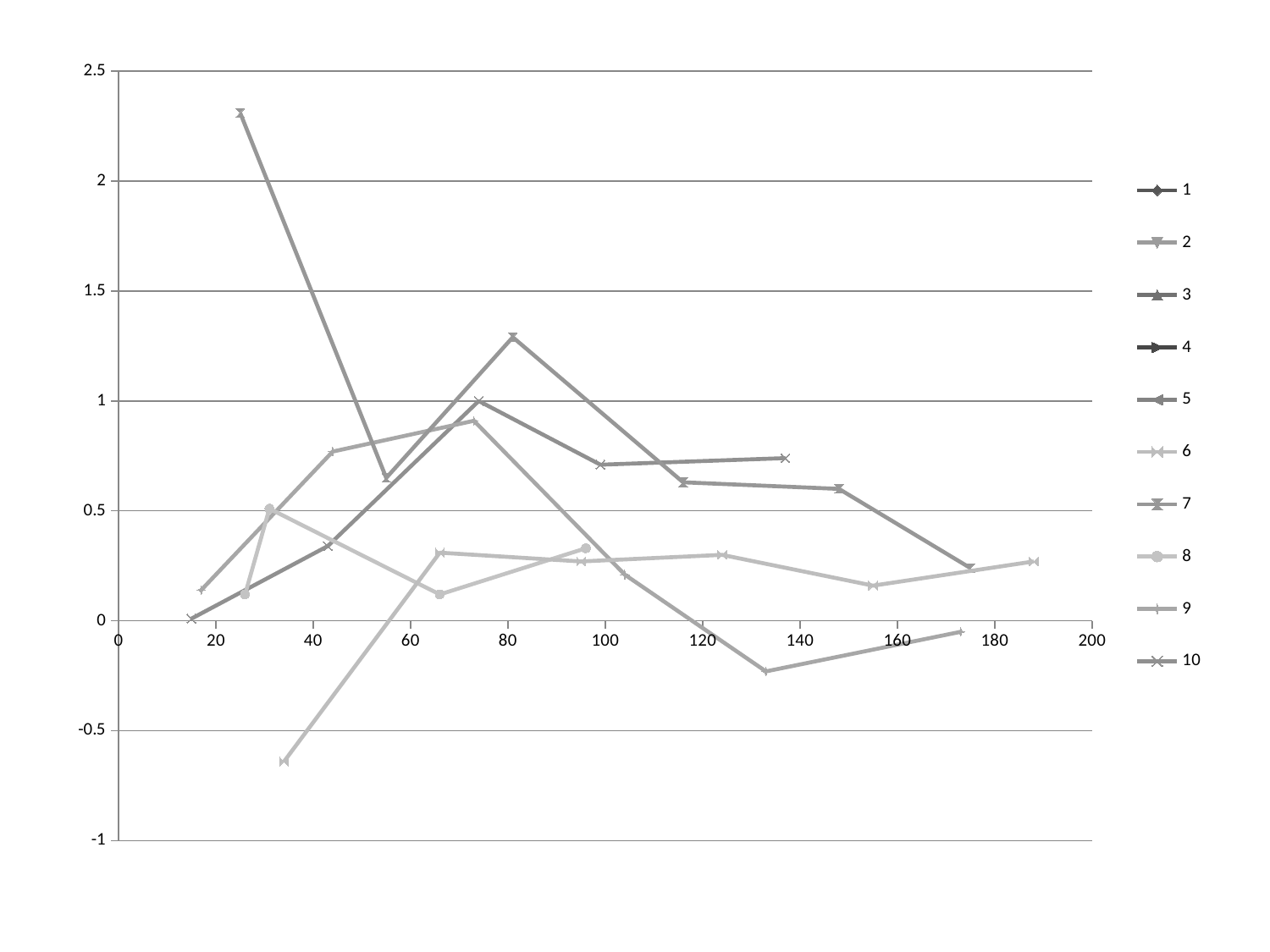

### Chart
| Category | | | | | | | | | | |
|---|---|---|---|---|---|---|---|---|---|---|

## Slide 2
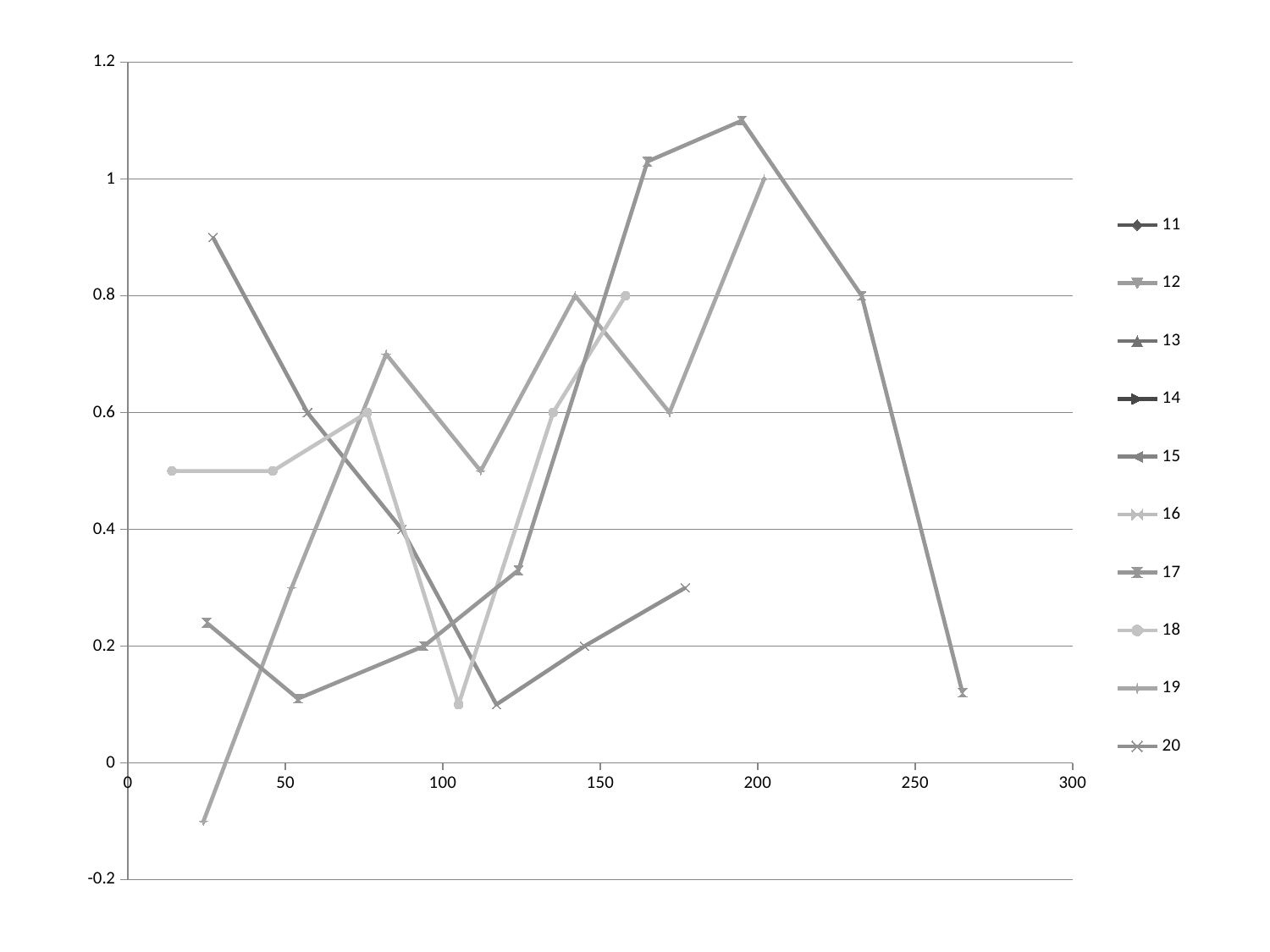

### Chart
| Category | | | | | | | | | | |
|---|---|---|---|---|---|---|---|---|---|---|

## Slide 3
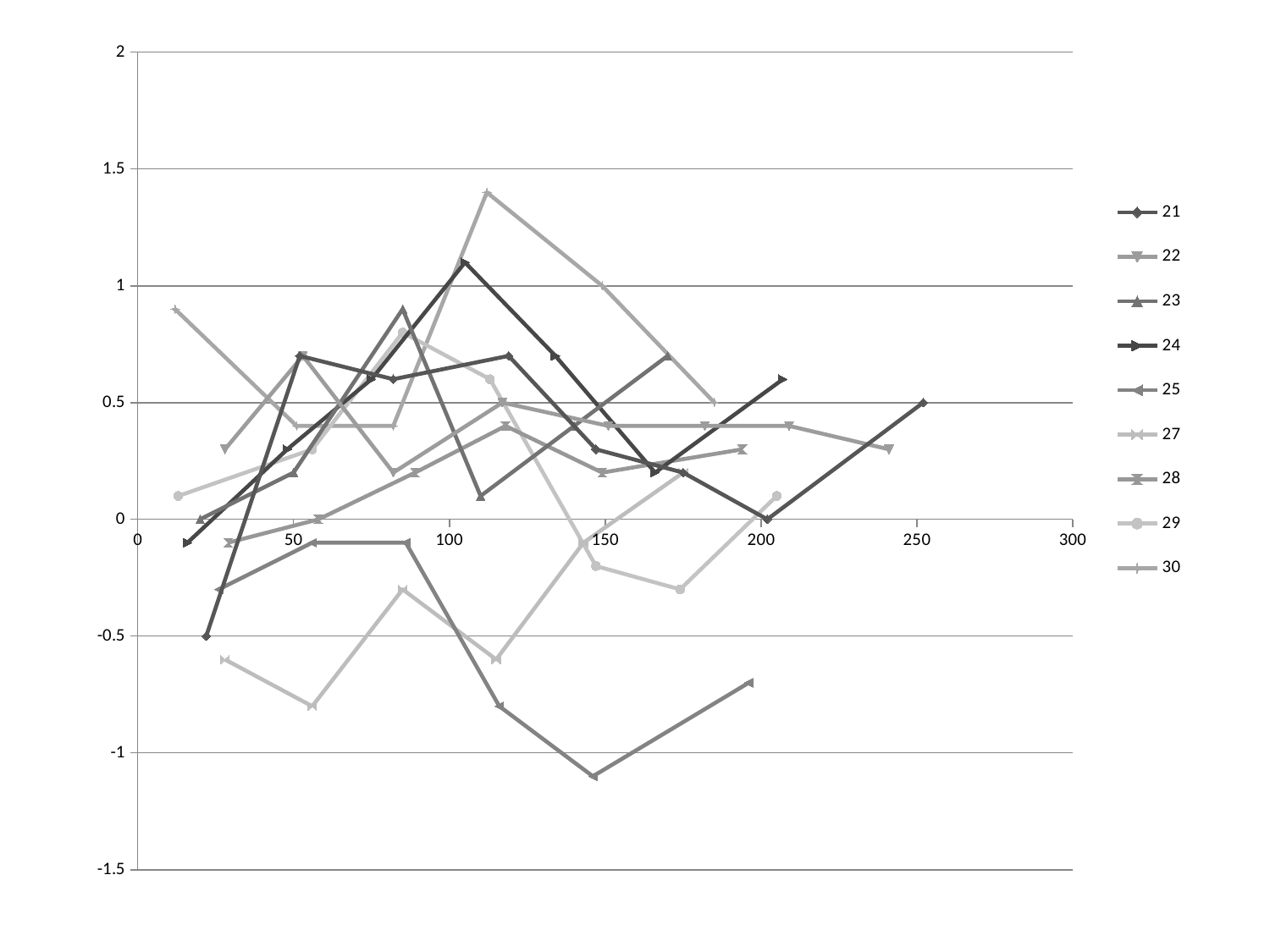

### Chart
| Category | | | | | | | | | |
|---|---|---|---|---|---|---|---|---|---|

## Slide 4
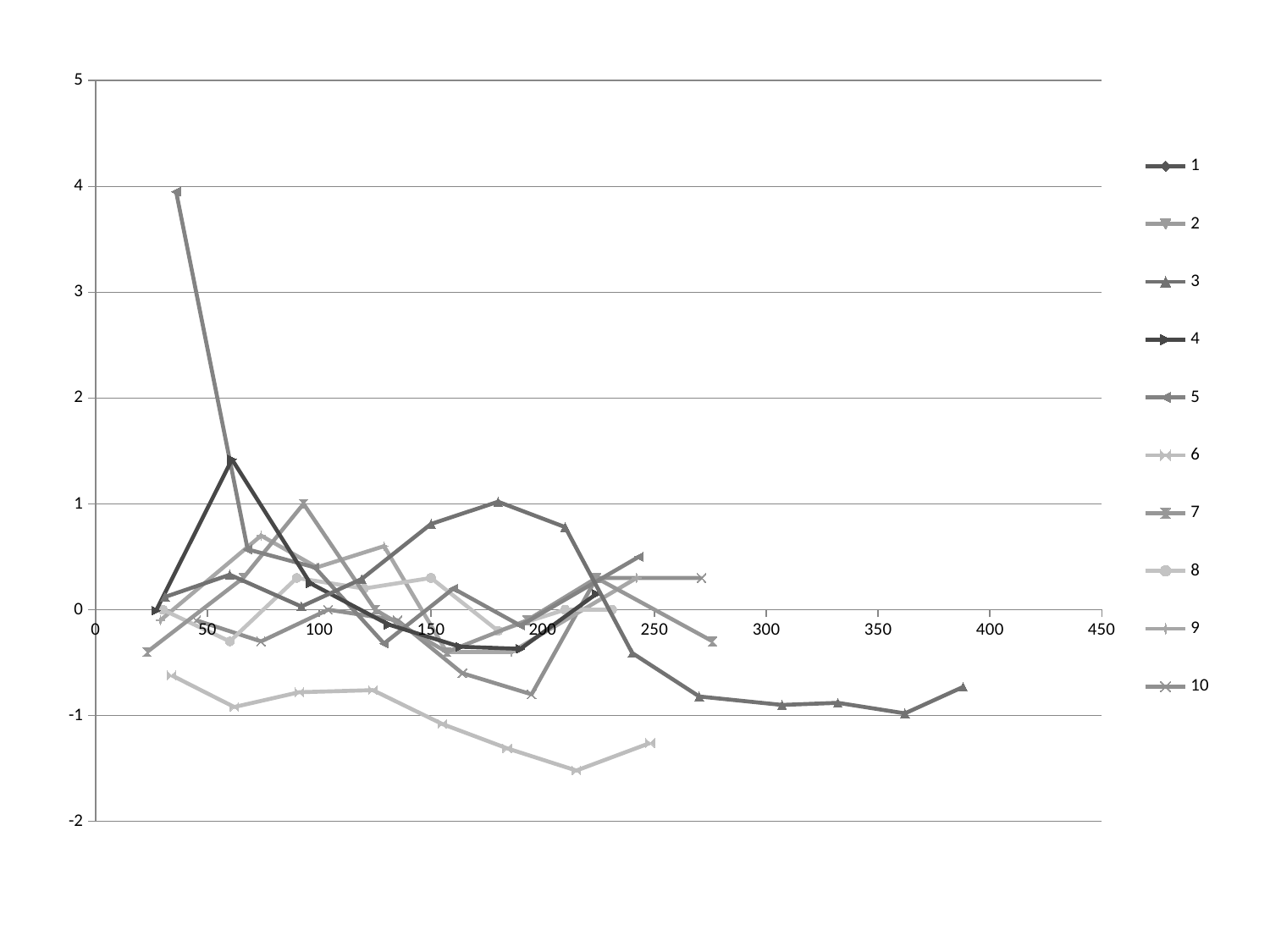

### Chart
| Category | | | | | | | | | | |
|---|---|---|---|---|---|---|---|---|---|---|

## Slide 5
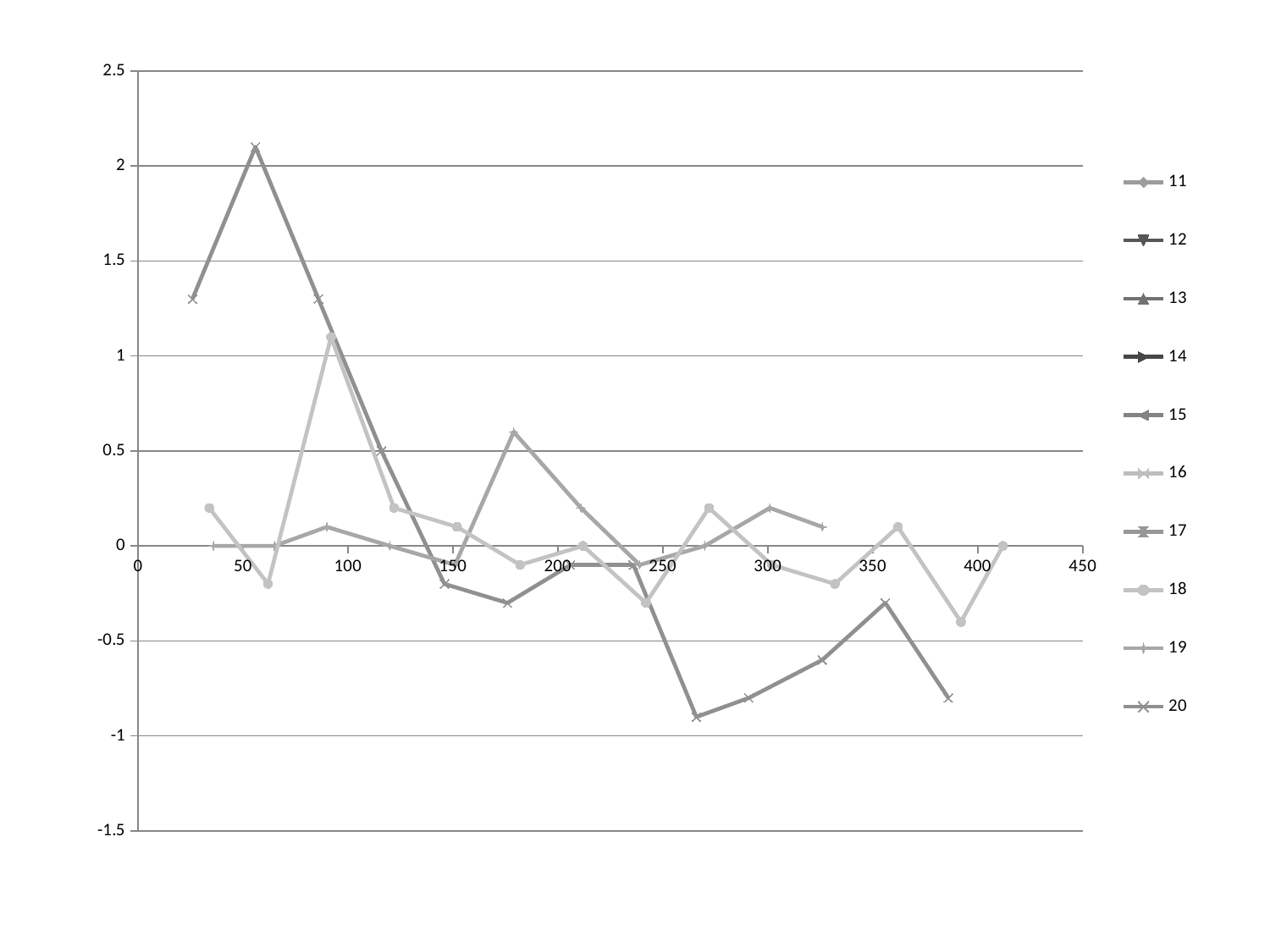

### Chart
| Category | | | | | | | | | | |
|---|---|---|---|---|---|---|---|---|---|---|
